# Physical frailty deteriorates after a 5‐day dexamethasone course in children with acute lymphoblastic leukemia, results of a national prospective study

**DOI:** 10.1002/cam4.6779

**Published:** 2023-12-09

**Authors:** Emma J. Verwaaijen, Annelienke M. van Hulst, Annelies Hartman, Rob Pieters, Marta Fiocco, Saskia M. F. Pluijm, Raphaële R. L. van Litsenburg, Martha A. Grootenhuis, Erica L. T. van den Akker, Marry M. van den Heuvel‐Eibrink

**Affiliations:** ^1^ Princess Máxima Center for Pediatric Oncology Utrecht The Netherlands; ^2^ Department of Pediatric Physiotherapy Erasmus Medical Center‐Sophia Children's Hospital Rotterdam The Netherlands; ^3^ Mathematical Institute Leiden University Leiden The Netherlands; ^4^ Department of Biomedical Data Science, Section Medical Statistics Leiden University Medical Center Leiden The Netherlands; ^5^ Department of Pediatrics, Division Pediatric Endocrinology, Obesity Center CGG Erasmus Medical Center‐Sophia Children's Hospital Rotterdam The Netherlands; ^6^ Division of Child Health Wilhelmina Children's Hospital Utrecht The Netherlands

**Keywords:** acute lymphoblastic leukemia, dexamethasone, frailty, muscle wasting

## Abstract

**Background:**

Dexamethasone is important in the treatment for pediatric acute lymphoblastic leukemia (ALL) but induces muscle atrophy with negative consequences for muscle mass, muscle strength, and functional abilities. The aim of this study was to establish the effect of a dexamethasone course on sarcopenia and physical frailty in children with ALL, and to explore prognostic factors.

**Methods:**

Patients with ALL aged 3–18 years were included during maintenance therapy. Patients had a sarcopenia/frailty assessment on the first day of (T1) and on the day after (T2) a 5‐day dexamethasone course. Sarcopenia was defined as low muscle strength in combination with low muscle mass. Prefrailty and frailty were defined as having two or ≥three of the following components, respectively: low muscle mass, low muscle strength, fatigue, slow walking speed, and low physical activity. Chi‐squared and paired *t*‐tests were used to assess differences between T1 and T2. Logistic regression models were estimated to explore patient‐ and therapy‐related prognostic factors for frailty on T2.

**Results:**

We included 105 patients, 61% were boys. Median age was 5.3 years (range: 3–18.8). At T1, sarcopenia, prefrailty, and frailty were observed in respectively 2.8%, 23.5%, and 4.2% of patients. At T2, the amount of patients with frailty had increased to 17.7% (*p* = 0.002), whereas the number of patients with sarcopenia and prefrailty remained similar. Higher ASMM (odds ratio [OR]: 0.49, 95% CI: 0.28–0.83), stronger handgrip strength (OR: 0.41, 95% CI: 0.22–0.77) and more physical activity minutes per day (OR: 0.98, 95% CI: 0.96–0.99) decreased the risk of frailty at T2. Slower walking performance (OR: 2, 95% CI: 1.2–3.39) increased the risk. Fatigue levels at T1 were not associated with frailty at T2.

**Conclusion:**

Physical frailty increased strikingly after a 5‐days dexamethasone course in children with ALL. Children with poor physical state at start of the dexamethasone course were more likely to be frail after the course.

## Graphical abstract

Dexamethasone is important in the treatment for pediatric acute lymphoblastic leukemia (ALL) but induces muscle atrophy. After a 5‐day dexamethasone course. Physical frailty increased with 13.5% in pediatric ALL patients. A poorer physical state at start of a dexamethasone course (lower muscle mass, muscle strength, or slower movement ability) was prognostic for developing frailty after a dexamethasone course.

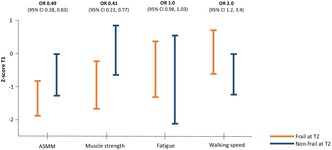

## 1 INTRODUCTION

Acute lymphoblastic leukemia (ALL) is the most common pediatric cancer worldwide, with a prevalence up to 25% of all cancers. Advances in treatment strategies and supportive care have resulted in a 5‐year survival rate of over 90% in high‐income countries.^1^, ^2^ However, children experience treatment‐related side effects which may interfere with physical abilities. These may include deterioration of muscle strength and muscle mass, which can be caused by malnutrition, infections, low physical activity, and by treatment with glucocorticoids.

In the general population sarcopenia, defined as the combination of low muscle mass and low muscle strength, is typically associated with (accelerated) aging.^3^, ^4^ However, there is growing evidence of this phenotype among children with ALL.^5^, ^6^ The same applies to the more extended vulnerability state: physical frailty. Frailty is characterized by three or more of these five components: low muscle mass, muscle weakness, self‐reported fatigue, slow walking speed, and low physical activity.^7^ Each of these components, muscle mass loss,^8^ muscle weakness,^9^, ^10^ fatigue,^11^, ^12^ slow walking speed,^10^ and reduced physical activity levels,^13^, ^14^ have individually been reported as side effects during treatment of ALL.

There is a partly overlap between sarcopenia and physical frailty, with both involving compromised muscle health.^15^ In the elderly, sarcopenia has been considered as a precursor of frailty,^16^, ^17^ but the biological and clinical relations between these two states are not yet clear.^15^ Nevertheless, as in older populations, sarcopenia, and frailty have both been associated with acute adverse health outcomes, that is, higher infection rates, increased hospitalizations, loss of ambulation, and even, impaired survival^6^, ^8^, ^18^, ^19^, ^20^ and with the onset of chronic comorbidities, disabilities, and early death in childhood cancer survivors.^2^

Dexamethasone is an important treatment component of ALL, but induces muscle atrophy of particularly Type II muscle fibers (which are the force generating fibers)^21^ and consequently myopathy.^22^ Among survivors of childhood cancer, higher cumulative doses of corticosteroids, and prolonged exposure have shown to increase muscle wasting and weakness.^15^

To date, it is not clear whether sarcopenia and frailty directly increase after a dexamethasone course during ALL treatment, and whether a child's initial physical state at the start of a course is prognostic for deterioration. Therefore, the primary objective of this study was to assess whether the frequency of sarcopenia and physical frailty, including its individual components increase after a 5‐day dexamethasone course. Second, we aimed to investigate whether patient‐ and/or treatment‐related factors, along with initial physical functioning, contribute to the development of frailty following a 5‐day dexamethasone course.

## 2 METHODS

### 2.1 Study design and cohort

This study on sarcopenia and physical frailty was performed within the framework of the DexaDays‐2 study: a national randomized controlled trial on neurobehavioral side effects of dexamethasone in pediatric ALL patients aged 3–18 years, conducted at the Princess Máxima Center for Pediatric Oncology, Utrecht, the Netherlands, between 2019 and 2021. The design of this study including in‐ and exclusion criteria has been previously described.^23^, ^24^

From 2011 tot 2020, children with ALL were treated according to the Dutch Childhood Oncology ALL‐11 protocol. In this protocol, patients were stratified to standard, medium, or high risk treatment. Medium risk (MR) maintenance treatment contained 28 three weekly treatment cycles. Patients received doxorubicin on the first day of the first 4 treatment cycles, vincristine once every 3 weeks, methotrexate once per week and 6‐mercaptopurine once per day, as well as dexamethasone for five consecutive days at the beginning of each treatment cycle (6 mg/m^2^ per day in three dosages). Depending on randomization, patients also received asparaginase once every 3 weeks until Week 15 or 27 of maintenance treatment.^25^ All participating patients had a sarcopenia/frailty assessment in the outpatient clinic, on the first day of a 5‐day dexamethasone course (T1) and on the day after this same course (T2). The assessment consisted of measurements of fatigue, muscle mass, muscle strength, and physical performance,^24^ and was carried out by a pediatric physiotherapist (EV) or medical physician (AvH) at the Sports and Exercise center of the Princess Máxima Center.

The study was approved by the Medical Ethics Committee (reference number NL62388.078.174) and all patients and/or parents provided written informed consent to participate.

### 2.2 Sarcopenia and frailty assessments

#### 2.2.1 Appendicular skeletal muscle mass

Appendicular skeletal muscle mass (ASMM), the sum of muscle mass of the four limbs, was measured using a multi‐frequency segmental bioimpedance analyzer (Tanita MC‐780, Tanita Corporation, Tokyo, Japan). As reference data for Dutch children were unavailable, to estimate SDS we used age and sex‐specific mean and standard deviation values from a UK population (5–18 years), acquired using the same Tanita software.^26^ Due to lack of bioimpedance reference values of 3‐ to 4‐year‐old children, we used sex and age specific expected values of ASMM (kilogram), derived by a dual‐energy x‐ray absorptiometry prediction equation in Canadian children^27^ (Table S1). Low ASMM was defined as SDS ‐1.5 or lower, in line with previous frailty studies.^2^, ^28^

#### 2.2.2 Muscle strength

Handgrip strength (kg) was measured in sitting position with the elbow flexed at 90^0^ using a hydraulic Jamar handheld dynamometer (Sammons Preston, Bolingbrook, Illinois, United States of America). For both the dominant and nondominant hand the mean score of three repeats was used. Mean values were compared to population‐based age and sex‐specific reference values and SDS^29^ were calculated. Low muscle strength was defined as SDS ‐1.5 or lower.

#### 2.2.3 Fatigue

The Dutch version of the Pediatric Quality of Life Inventory (PedsQL)—Multidimensional Fatigue Scale (MFS) was used to assess fatigue‐related problems.^30^ This questionnaire consists of three scales: general fatigue, sleep/rest fatigue, and cognitive fatigue. We used the parental versions for the specific age groups 3–4, 5–7, 8–12, and 13–18 years. Subsequently, we compared total scores of our population to Dutch reference values and calculated SDS.^30^ Patients with a SDS of −1.5 or lower were classified as fatigued.

#### 2.2.4 Walking speed

The Timed Up and Go test (TUG) was used to asses walking speed. The children started seated on a chair and were asked to stand up, walk 3 m, turn around, walk back, and sit down again. The mean time of three attempts was considered as the test result, and SDS were calculated using a Brazilian age and weight specific reference equation.^31^ Patients with a SDS of 1.5 or higher were classified as slow (higher SDS indicates lengthier and thus slower performance).

#### 2.2.5 Physical activity

Physical activity was assessed using questionnaires. For children 3–11 years of age we used parent proxy‐reported questionnaires generated in a Dutch population‐based prospective cohort study.^32^ These questionnaires contained questions regarding frequency and duration of outdoor playing, sports participation and active commuting to/from school. Time per week spent on each activity was calculated by using the following equation: weekly time spent on the activity = (days per week) × (hours per day). Total physical activity was calculated by adding the hours of active commuting, outdoor play, and sport participation per week. For the definition of low physical activity, we used a cutoff of less than 60 active minutes per day, based on the World Health Organization guidelines for physical activity.^33^

Children 12–18 years were asked to fill in the modified Baecke questionnaire.^34^ Physical activity during school, leisure time, and organized sports were reported in frequency, intensity, and duration. Total physical activity was calculated according to the Baecke formula.^35^ As a reference cohort we used Baecke scores reported in 102 Dutch children, 10.5 ± 3.6 years of age.^36^ We defined low physical activity in this study as a score 1.5 SDS below their reported mean score.

A complete overview of the measuring instruments including methods and psychometric properties have previously been described in our study protocol.^24^

#### 2.2.6 Definition of sarcopenia and physical frailty

Sarcopenia was defined as the combination of low muscle strength and low muscle mass.^3^ Prefrailty and frailty were classified as the presence of respectively two, or more than two of the following five components: low muscle mass, low muscle strength, fatigue, slow walking speed, and low physical activity.^7^ Figure S1 provides an overview of the two phenotypes and the overlap between the definition and the individual components.

### 2.3 Potential prognostic factors for frailty

The following variables were assessed as prognostic factors for frailty after 5 days of dexamethasone administration: sex, age in years, weight SDS, body mass index (BMI) SDS, maintenance week, concomitant asparaginase (depending on ALL‐11 randomization children received asparaginase until Week 15 or 27 of maintenance therapy). In addition, we assessed if the physical functioning state at T1 was associated with the occurrence frailty at T2. These outcomes were: ASMM SDS, handgrip strength SDS, PedsQL‐MFS SDS (fatigue), TUG SDS (rising/walking speed) and physical activity minutes per day. The latter only applied to children aged 3–11 years.

### 2.4 Statistics

Patient characteristics and assessment results were presented as mean or median with interquartile range (IQR), according to the distribution of the variables. Paired *t*‐test was used to assess differences in test results between at start (T1) and after 5‐days (T2) of the dexamethasone course. In case of violation of the normality assumption, Wilcoxon ranked sum test was employed. Mean/median differences with 95% confidence interval in raw scores and SDS were reported. Chi‐squared and Fisher's exact tests were used to compare the occurrence of sarcopenia and frailty and the individual components (low ASMM, low muscle strength, fatigue, slow walking speed, and low physical activity) at T1 and T2. To investigate potential prognostic factors (patient‐, disease‐, and therapy‐related characteristics and T1 assessment results) for frailty at T2, univariable logistic regression models were estimated. Odds ratio (OR) was not estimated when the number of participants in a cell of the contingency table was ≤3. All analyses were performed in R software environment Version 1.4.1106 for Windows.

## 3 RESULTS

### 3.1 Patients

**FIGURE 1:**
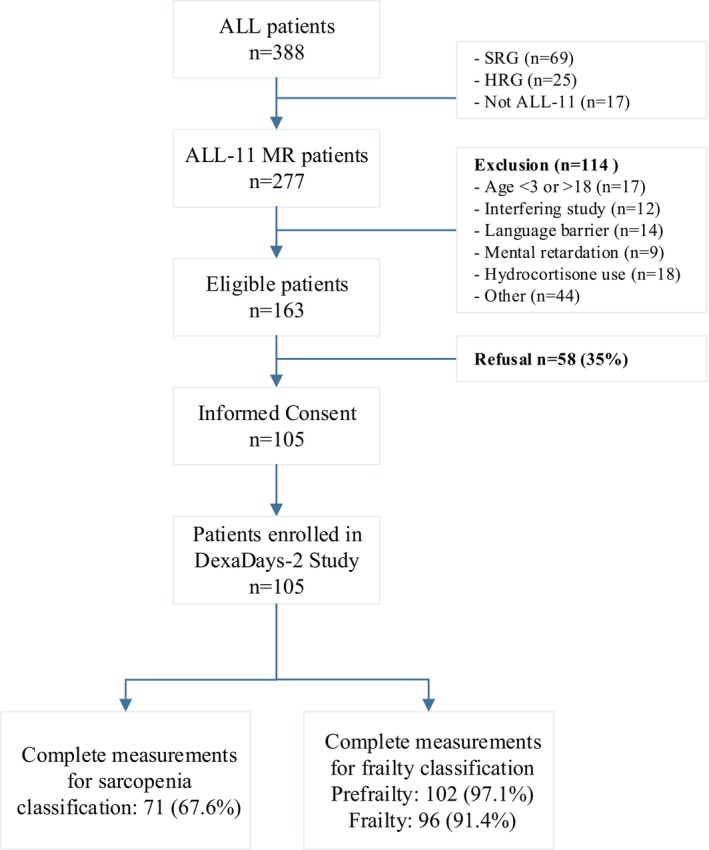
Flowchart of study patients and completed assessments.

In total, 105 patients with ALL undergoing MRG maintenance therapy were included in this study (Figure 1: Flowchart) with a median age of 5.3 years (range: 3–18.8). The majority were boys (61%). Ninety‐three patients (88.6%) had pre B‐cell ALL, 11 patients (10.5%) had T‐cell ALL and one patient had a blastic plasmacytoid dendritic cell neoplasm but was also treated according to the DCOG ALL‐11 MRG protocol and therefore included (Table 1).

**TABLE 1:** Patient characteristics (*N* = 105).

	Median	IQR	Range
Age, years	5.33	4.17, 8.83	3.0, 18.83
Weight, SDS^a^	0.34	1.27	−3.62, 4.41
Height, SDS^a^	−0.83	1.05	−3.18, 1.56
Body mass index, SDS^a^	1.11	1.12	−3.28, 3.88
Maintenance week, number	34	22, 43	13, 68

	No.	%	
Sex			
Female	41	39	
Male	64	61	
Type of ALL			
Pre‐B ALL	93	88.6	
T‐ALL	11	10.5	
BPDCN^b^	1	0.9	

Abbreviations: ALL, acute lymphoblastic leukemia; BPDCN, blastic plasmacytoid dendritic cell neoplasm; SDS, Standardized deviation score.

^a^ SDS values are mean with standard deviation.

^b^ One patient had BPDCN and was also treated according to the ALL‐11 protocol.

### 3.2 Sarcopenia and physical frailty (components) before and after 5‐day dexamethasone course (T1‐T2)

At T1, sarcopenia was present in 2 (2.8%) patients, while prefrailty and frailty were observed in 24 (23.5%) and 4 (4.2%) patients, respectively. At T2, the number of patients with sarcopenia and prefrailty remained similar, while the prevalence of frailty increased with 13.5%–17.7% (*p* = 0.002). Complete assessment results and paired analyses are depicted in Table 2.

**TABLE 2:** Assessment results and paired analyses of the frailty components on Day 1 (T1) and 1 day after (T2) a 5‐day dexamethasone course.

		Day 1		Day 6		Paired difference Day 1–6	
	*N*	Mean	IQR	Mean	IQR	Mean Δ	95% CI
Weight, kg	86	21.6^a^	28.7, 37.5	22.1^a^	18.5, 37.9	−0.05	−0.2 to 0.1
Appendicular skeletal muscle mass	83						
Kilogram		5.4^a^	4, 10.5	5^a^	3.8, 10	**−0.55** ^b^	**−0.7 to −0.45**
Percentage		25.03	21.71, 27.24	23.06	20.25, 25.82	**−1.97**	**−2.3 to −1.65**
SDS		−0.65	−1.37, −0.08	−1.19	−1.88, −0.59	**−0.54**	**−0.65 to −0.44**
Muscle strength							
Handgrip strength, dominant hand	82						
Kilogram		9.15^a^	6.35, 14.7	10.1^a^	7, 16	**0.65** ^b^	**0.25 to 1.05**
SDS		−0.02	−0.7, 0.8	0.22	−0.7, 0.98	**0.24**	**0.13 to 0.35**
Fatigue							
PedsQL—MFS	92						
Total score		76.39^a^	58.33, 87.85	41.67	31.6, 57.29	**−24.31** ^b^	**−29.17 to −19.44**
SDS		−0.55	−2.18, 0.53	−3.17	−4.6, −2.04	**−2.13**	**−2.54 to −1.72**
Slow walking speed							
Timed Up and Go test	91						
Seconds		5.62	4.58, 6.44	5.63	4.55, 6.33	0.02	−0.19 to 0.23
SDS		−0.47	−1.25, 0.13	−0.42	−1.33, 0.07	0.05	−0.14 to 0.24
Physical activity							
3–11 years—questionnaire	72						
Active minutes per day		83.57^a^	44.46, 120.18	49.29^a^	15, 103.57	**−26.79** ^b^	**−39.64 to −15**
12–18 years—Baecke questionnaire	6						
Total score		7.24^a^	6.32, 9.01	6.27^a^	5.17, 8.38	−0.56^b^	−1.45 to 0.14
SDS		−1.71^a^	−2.88, −0.27	−2.93^a^	−4, −0.88	−0.54^b^	−1.41 to –0.14

		No.	%	No.	%		*p*‐value
Frailty components							
Low skeletal muscle mass	83	20	24.1	32	38.6		**0.003**
Low handgrip strength	82	10	12.2	8	9.76		0.683
Fatigue	92	35	38.04	76	82.61		**<0.00001**
Slow walking speed	91	4	4.4	6	6.59		0.683
Low physical activity	78	31	39.74	45	57.69		**0.004**
Sarcopenia	71	2	2.8	2	2.8		0.999
Prefrailty	102	24	23.5	29	28.4		0.458
Frailty	96	4	4.2	17	17.7		**0.002**

Abbreviations: CI, confidence interval; IQR, interquartile range; SDS, standardized deviation score.

^a^ Median.

^b^ Median value based on Wilcoxon signed rank test. Bold indicates statistical significance.

At T1, Mean ASMM was 25% (IQR: 21.7, 27.2), which was lower compared to normative values (SDS: −0.65, IQR: −0.37, −0.08). Twenty (24.1%) patients were classified as having low ASMM. At T2, ASMM had decreased with −0.54 SDS (95% CI: −0.65, −0.44), while body weight remained unchanged (SDS: −0.05, 95% CI: −0.2, 0.1). There was a significant 14.5% increase in the prevalence of patients with low ASMM (*p* < 0.01).

At T1, handgrip strength was within normal ranges (SDS: ‐0.02, IQR: −0.7, 0.8). Ten (12.2%) patients had low strength. Handgrip strength had improved with 0.2 SDS (95% CI: 0.1, 0.4) at T2, but the number of children with low strength remained the same (*p* = 0.68).

Median total PedsQL‐MFS score was 76.4 (IQR: 58.3, 87.9) at T1. Compared to normative values, mean SDS was −0.55 (IQR: −2.2, 0.5) and 35 (38%) children were classified as fatigued. At T2, the PedsQL‐MFS total score had decreased by −2.13 SDS (95% CI: −2.54, −1.72), resulting in a 44.6% increase of fatigued patients (*p* < 0.01).

The mean TUG time was 5.6 s (IQR: 4.6, 6.4) at T1, which was lower compared to normative values (SDS: ‐0.5, IQR: −1.3, 0.1). Four (4.4%) children had a low score, which was similar at T2 (*p* = 0.68).

At T1, children 3–11 years were on average 83.6 min active per day (IQR: 44.5, 120.2). Older children and adolescents (12–18 years, n = 6) had a Baecke physical activity SDS of −1.7 (IQR: −2.9, −0.3). In total, 31 patients (39.7%) met the criteria for low physical activity. At T2, In 3‐ to 11‐ year‐olds physical activity decreased with −26.8 minutes per day (95% CI: −39.6, −15). The Baecke SDS showed a decrease of −0.54 (95% CI: −1.41, 0.14) in patients 12–18 years. The number of patients with low physical activity had increased with 18% (*p* < 0.01).

**FIGURE 2:**
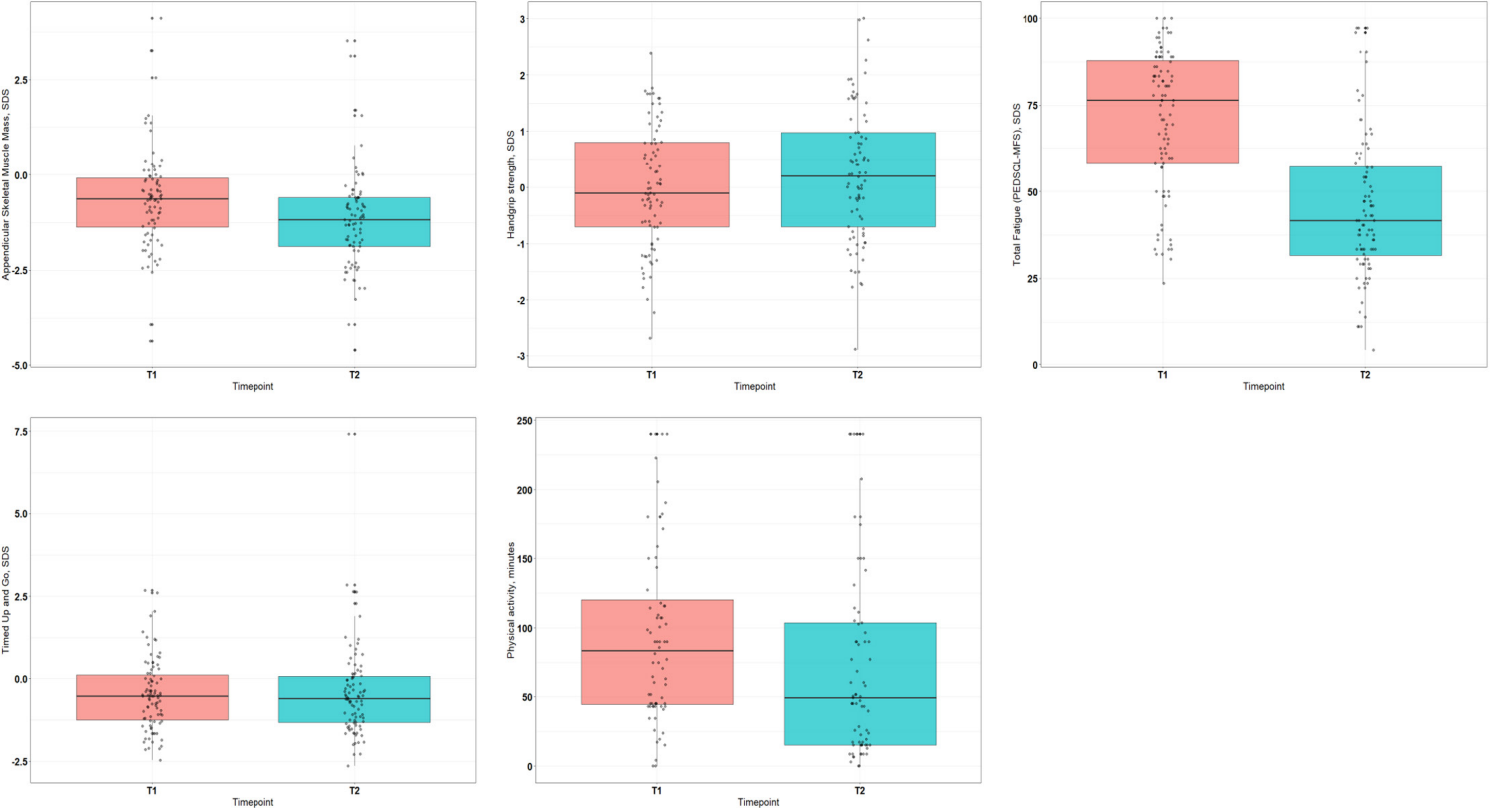
Boxplots visualizing the score differences between the first day (T1) and sixth day (T2) of dexamethasone administration.

The differences in mean scores are visualized Figure 2.

### 3.3 Determinants for frailty after a 5‐day dexamethasone course (T2)

Seventeen patients were classified as physically frail at T2. Univariable logistic regression models showed that lower weight SDS at T1 was negatively associated with frailty at T2 (OR: 0.54, 95% CI: 0.33–0.89) (Table 3). Patients who were further into maintenance therapy were less prone to become frail (OR: 0.94, 95% CI: 0.9–0.98). Concomitant administration of asparaginase did not have a negative effect on developing frailty but the number of children still receiving asparaginase was relatively small (*n* = 11) (OR: 3.12, 95% CI: 0.8–12.2).

**TABLE 3:** Univariable association between determinants at Day 1 (T1) and having frailty at Day 6 (T2).

Determinants at T1	Frail at T2	Non‐Frail at T2	Odds ratio	95% CI
Sex			1.0	0.35–2.92
Female	7 (41.2)	32 (41)		
Male	10 (58.8)	46 (59)		
Age, years	6.4 (4, 9)	5.3 (4.25, 8.85)	1.0	0.9–1.14
Weight, SDS	−0.34 (−0.83, 0.32)	0.43 (−0.31, 1.24)	**0.54**	**0.33–0.89**
Body mass index, SDS	−1.41 (−1.78, −0.8)	−0.74 (1.53, 0.07)	0.53	0.27–1.03
Type of ALL			N.a.	
Pre B‐cell	14 (82.3)	71 (91)		
T‐cell	2 (11.8)	7 (9)		
BPDCN	1 (5.9)	0 (0)		
Maintenance week, number	25 (16, 31)	37 (25.5, 46)	**0.94**	**0.9–0.98**
Appendicular skeletal muscle mass, SDS	−1.39 (−1.84, −0.87)	−0.54 (−1.28, −0.02)	**0.49**	**0.28–0.83**
Dominant handgrip strength, SDS	−0.73 (−1.65, 0.2)	0 (−0.6, 0.88)	**0.41**	**0.22–0.77**
Fatigue (PedsQL‐MFS), SDS	−0.46 (−1.31, 0.38)	−0.49 (−2.1, 0.56)	1.0	0.98–1.03
Timed Up and Go test, SDS	0.26 (−0.52, 0.68)	−0.54 (−1.28, −0.02)	**2.02** ^b^	**1.2–3.39**
Physical activity, minutes per day^a^	44 (23, 62)	90 (45, 147)	**0.98**	**0.96–0.99**

*Note*: Values are depicted as median (interquartile range) or number (%), Bold indicates statistical significance.

Abbreviations: BPDCN, blastic plasmacytoid dendritic cell neoplasm, n.a., not applicable.

^a^ Analyzed in subcohort of children 3–11 years of whom physical activity minutes per day were available (*n* = 72).

^b^ Higher SDS = slower performance increases the risk of frailty.

**FIGURE 3:**
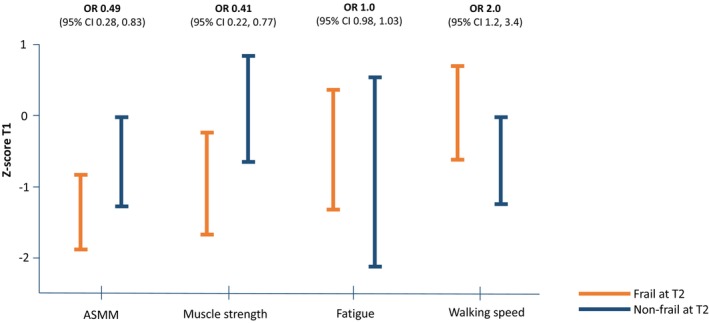
The difference in appendicular skeletal muscle mass (ASMM), muscle strength, fatigue level and walking speed at T1, between patients that were frail and non‐frail at T2. T1, at start of a 5‐day dexamethasone course; T2, 1 day after completion of the same course.

Poor physical status and performance at T1 were associated with a higher frailty occurrence at T2 (Figure 3). Higher ASMM (OR: 0.49, 95% CI: 0.28–0.83), stronger handgrip strength (OR: 0.41, 95% CI: 0.22–0.77) and more physical activity minutes per day (OR: 0.98 95% CI: 0.96–0.99) decreased the risk of frailty at T2 significantly. Slower performance on the TUG (OR: 2, 95% CI: 1.2–3.39) increased the risk. Fatigue levels at T1 were not associated with frailty at T2.

## 4 DISCUSSION

Our study showed that the occurrence of physical frailty increased with 13.5% directly after a 5‐day dexamethasone course in children with ALL. This is a concerning finding as dexamethasone pulses are recurrently administered in many ALL chemotherapy schedules, and a physical frail state has been associated with an increased risk of adverse events in pediatric populations.^19^, ^20^

There was a notably smaller percentage of patients with sarcopenia (patients who had both low muscle mass and muscle strength) (2.8%) and this occurrence was not increased after a dexamethasone course. As ASMM did decrease during the course, this may indicate that a decrease in strength does not manifest as acutely, or it could be that handgrip strength may not reflect total muscle strength in these young patients.

We did find a marked decrease of −0.5 SDS in ASMM after 5 days of dexamethasone administration, which may be related to the catabolic effect of dexamethasone.^37^ However, the acute effect of dexamethasone administration and the role of pharmacokinetics in muscle deterioration needs to be studied in further depth. The decrease in ASMM could also potentially explained by the striking observed decline in physical activity (on average 27 min less physically active per day). Studies in healthy young, and older adults indicated that short‐term sedentary behavior already led to significant loss of skeletal muscle mass.^38^, ^39^ It is not known whether this effect of muscle breakdown in children is also this profound. Although ASMM decreased during the dexamethasone course, total body weight remained the same which may be explained by fluid imbalances or fat increase (cushingoid features). Our analyses also showed that lower body weight SDS and lower ASMM SDS at T1 were associated with frailty at T2, unlike BMI SDS, which was unexpectedly not associated.

Parents reported a dramatic increase in their children's fatigue after 5 days of dexamethasone administration. At day one, 38% already had a high fatigue score but this increased to 83% of the children on Day 6. This increase is consistent with a previous study in children with ALL.^11^, ^40^ The precise mechanism behind fatigue during dexamethasone treatment has not been elucidated yet.^41^ Although fatigue was the component that showed the largest increase after 5 days of dexamethasone, this was the only component at T1 that was not prognostic for frailty at T2, suggesting that perceived fatigue is not associated with decline in muscle and function. Nonetheless, fatigue is a striking problem for which there are no standardized effective interventions available yet, although previous studies indicated that exercise interventions may be promising. In a small controlled trial (*n* = 22), a 6‐week homebased aerobic exercise intervention during ALL maintenance therapy showed reductions in fatigue.^42^ In a longitudinal observation of 68 children with various types of cancer increased physical activity levels were associated with less fatigue.^43^ Another pilot study (*n* = 17) showed that children with the highest step counts in the week before the corticosteroid course, reported less fatigue during the corticosteroid course.^44^ However, it is unclear if these results indicates a causal relationship or co‐association. Moreover, in adolescents without cancer, cognitive behavior therapy has been shown to be a successful intervention in reducing severe fatigue.^45^ In children with cancer there has been only one noncontrolled pilot study so far, which does show promising results.^46^

Somewhat surprisingly, patients did not reveal a decline in handgrip strength and movement speed. We even reported an increase in handgrip strength SDS. However, we suspect this improvement may have been based on a learning effect (repetition of the measurement within 5 days may have had a beneficial effect on performance). We are also hesitant about whether the instruction (squeezing as hard as possible) can be performed properly by 3‐ and 4‐year‐olds. We expected to observe a decline in both handgrip strength and walking speed, partly because of the co‐administration of vincristine at T1. Vincristine is known to induce peripheral neuropathy with consequent strength loss in distal muscles and clumsiness.^47^

Since dexamethasone courses are repeated 28 times (every 3 weeks for 1.5 years) during ALL maintenance therapy, it is conceivable that the repetitive impact of dexamethasone treatment may change over time. We observed that children who were further into maintenance therapy (rather than newly started), were less often frail. Patients early in maintenance phase, may not have entirely recovered from the intensive induction and consolidation phase (high doses of chemotherapy, immobilization, and infections) or there may be a nuisance effect of asparaginase administration. Asparaginase has also been implicated as a potential contributor to reduced muscle health,^15^ but the exact mechanism is currently unknown. Asparaginase has an inhibitory effect on protein synthesis in cancer cells, it is hypothesized that muscle protein synthesis in muscle cells may also be compromised.^15^ As our study had only 11 children who still received asparaginase, we were not able to analyze this thoroughly.

This is the first prospective study to assess the acute effect of a dexamethasone course on the individual components of sarcopenia and physical frailty in a national cohort of children with ALL. We showed that a patient's muscle mass, muscle strength and physical performance, before the start of a dexamethasone course, is prognostic for developing frailty after the course. This finding may endorse the “better in and better out principle,” and gives us reason to explore specific interventions for dexamethasone resilience, to prepare our patients for dexamethasone courses. Current evidence for interventions to improve muscle mass and function in children with cancer is not very comprehensive. From a biological perspective it is hypothesized that exercise interventions potentially increase repair of ‐by chemotherapy‐ damaged mitochondria.^48^ However, only a number of exercise trials have been performed and showed mixed success in effectiveness, but did show that exercise is safe and feasible even during intensive treatment.^49^, ^50^, ^51^ For future research, we aim to determine the most beneficial training and right timing for the individual patient, that is, whether structured aerobic exercise, resistance training or only a higher level of physical activity (increased step count) will yield positive results on muscle health. Moreover, further knowledge and deep understanding of frailty in pediatric cancer patients is needed to develop successful interventions.

This study has some limitations to be addressed. First, we used bioimpedance analysis to assess ASMM, which is a safe, cost‐efficient, and quick method. The downside is uncertainty about the reliability of bioimpedance analyses in children with high fat percentages,^52^ and also hydration status may affect the measurements, as it causes an increase in the body's electrical resistance.^53^ Both overweight and disturbed fluid balance can occur in ALL patients, thus this may have influenced our results. However, current reliable imaging techniques such as computed tomography (unsuitable due to radiation exposure), magnetic resonance imaging and dual‐energy x‐ray absorptiometry are expensive, poor accessible and time consuming. Second, we had no availability of Dutch normative values for the used measurement instruments (besides PedsQL‐MFS). Therefore, we used pediatric reference values from cohorts with other origins, which may have influenced our results. Third, the current findings can only be generalized specifically to protocols that involve the administration of maintenance dexamethasone for a duration of 5 days, which is currently within the ALLTogether protocol across 14 different European countries. Fourth, while we observed an association between patients further into maintenance therapy and a reduced risk of frailty, we were unable to unravel this finding in relation with complications and adverse events occurring earlier in treatment, due to limited data collection during those phases in this cohort. We recommend that these factors be considered in future studies.

In conclusion, 5 days of dexamethasone increased physical frailty in children with ALL. A poorer physical state at start of a dexamethasone course (lower muscle mass, muscle strength, and slower movement ability) was prognostic for developing frailty after a dexamethasone course.

## AUTHOR CONTRIBUTIONS

**Emma J. Verwaaijen:** Data curation (equal); formal analysis (equal); methodology (equal); project administration (equal); writing – original draft (lead). **Annelienke M. van Hulst:** Data curation (equal); investigation (equal); project administration (equal); writing – review and editing (equal). **Annelies Hartman:** Supervision (equal); writing – review and editing (equal). **Rob Pieters:** Conceptualization (equal); methodology (equal); resources (supporting); supervision (lead); writing – review and editing (equal). **Marta Fiocco:** Formal analysis (equal); methodology (lead); writing – review and editing (equal). **Saskia M. F. Pluijm:** Conceptualization (supporting); methodology (supporting); writing – review and editing (supporting). **Raphaële R. Van Litsenburg:** Methodology (supporting); writing – review and editing (supporting). **Martha A. Grootenhuis:** Conceptualization (equal); funding acquisition (equal); resources (equal); supervision (equal); writing – review and editing (equal). **Erica van den Akker:** Conceptualization (equal); methodology (equal); supervision (equal); writing – review and editing (equal). **Marry M. van den Heuvel‐Eibrink:** Conceptualization (lead); resources (lead); supervision (lead); writing – review and editing (lead).

## FUNDING INFORMATION

Stichting Kinderen Kankervrij (KiKa): projectnumber 268.

Stichting de Wonderlijke reis.

## ETHICS STATEMENT

The study was approved by the Medical Ethics Committee (reference number NL62388.078.174).

## Supporting information


Figure S1.

Table S1.

## Data Availability

The data that support the findings of this study are available from the corresponding author [EJV] upon reasonable request.
