# Supplementary material for: Physical frailty deteriorates after a 5‐day dexamethasone course in children with acute lymphoblastic leukemia, results of a national prospective study
Source: Cancer Med. 2023 Dec 9;12(24):22304–15. doi: 10.1002/cam4.6779 (PMC10757098; doi:10.1002/cam4.6779)
Supplement: Supplementary file 1 — Figure S1. [file CAM4-12-22304-s001.pdf]

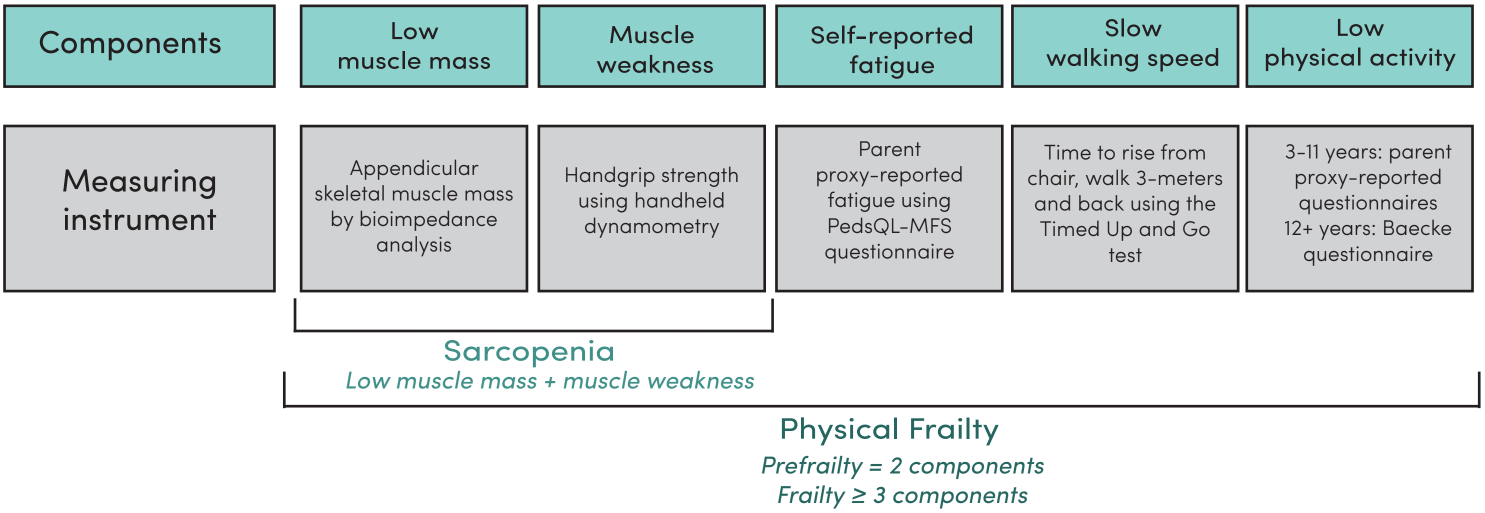
Components
Low muscle mass
Muscle weakness
Self-reported fatigue
Slow walking speed
Low physical activity
Measuring instrument
Appendicular skeletal muscle mass by bioimpedance analysis
Handgrip strength using handheld dynamometry
Parent proxy-reported fatigue using PedsQL-MFS questionnaire
Time to rise from chair, walk 3-meters and back using the Timed Up and Go test
3-11 years: parent proxy-reported questionnaires
12+ years: Baecke questionnaire
Sarcopenia
Low muscle mass + muscle weakness
Physical Frailty
Prefrailty = 2 components
Frailty ≥ 3 components
