# Supplementary material for: Physical frailty deteriorates after a 5‐day dexamethasone course in children with acute lymphoblastic leukemia, results of a national prospective study
Source: Cancer Med. 2023 Dec 9;12(24):22304–15. doi: 10.1002/cam4.6779 (PMC10757098; doi:10.1002/cam4.6779)
Supplement: Supplementary file 2 — Table S1. [file CAM4-12-22304-s002.docx]

| **Supplemental Table 1. Appendicular skeletal muscle mass values in the DexaDays-2 cohort, and UK and Canadian reference values** | | | | | | | | | | | |
| --- | --- | --- | --- | --- | --- | --- | --- | --- | --- | --- | --- |
|  | **Dutch ALL cohort**  **(Tanita bio-impedance analysis at T1)** | | | | | **UK reference values^1^**  **(Tanita bio-impedance analysis)** | | | | **Predicted ASMM based on Canadian DXA reference equation^2^** | |
| **Boys** |  | | | | |  | | | |  | |
| **Age** | **N** | **BMI, kg/m^2^** | **ASMM, kg** | **95% CI** | **ASMM, %** | **N** | **BMI, kg/m^2^** | **ASMM, kg** | **ASMM, %** | **ASMM, kg** | **95% CI** |
| 3-4 years | 22 | 17.1 (1.3) | 3.5 | 2.4 – 6.3 | 20.1 (3.3) |  |  |  |  | 4.3 | 2.5 - 6.8 |
| 5–7 years | 15 | 16.4 (1.4) | 5.2 | 3.2 – 8.4 | 23.7 (3.6) | 329 | 15.4 (2.3) | 6.3 (1.8) | 27.2 (2.9) | 7.6 | 4.1 – 11.6 |
| 8–10 years | 7 | 18.8 (2.6) | 10.2 | 6.2 – 13.6 | 28.5 (4.4) | 296 | 17.5 (3.3) | 10 (2.9) | 29.6 (5.1) | 11.3 | 6.1 – 17.5 |
| 11–13 years | 5 | 17.3 (0.9) | 11.6 | 10.2 - 14 | 29.8 (1.6) | 204 | 19 (3.2) | 14.8 (3.8) | 32.6 (3.4) | 15.4 | 7.7 – 23.1 |
| 14–16 years | 6 | 23.2 (6.2) | 21.9 | 17.1 – 30.6 | 31.8 (4.1) | 149 | 20.4 (2.7) | 21.7 (4.7) | 35.3 (4.8) | 26.2 | 10.1 – 38.4 |
| 17-18 years | 3 | 24.5 (6.4) | 27.4 | 22.2 – 30.5 | 32.9 (3.7) | 138 | 22.4 (2.8) | 24.8 (4.3) | 34.9 (4.8) | 31.6 | 11.8 – 43.4 |
| **Girls** |  |  |  |  |  |  |  |  |  |  |  |
| 3-4 years | 16 | 16.8 (1.8) | 4 | 2.6 – 5.7 | 23.3 (2.7) |  |  |  |  | 4.1 | 2.6 – 5.6 |
| 5–7 years | 14 | 16.8 (2) | 5.3 | 4.1 – 7.5 | 24.1 (2.2) | 217 | 15.4 (2.3) | 6.1 (1.4) | 27.1 (2.4) | 5.8 | 3.7 - 8 |
| 8–10 years | 5 | 19.5 (1.4) | 9.3 | 5.9 – 10.5 | 25.6 (3) | 157 | 18 (3.6) | 9.1 (2.5) | 27.1 (4.2) | 8.3 | 5.1 – 11.3 |
| 11–13 years | 2 | 16.8 (0.4) | 9.8 | 8.2 – 11.2 | 26.5 (2.5) | 199 | 19.2 (3.1) | 12.8 (3) | 28.3 (4.3) | 14.2 | 10.2 – 18.2 |
| 14–16 years | 1 | 24.3 | 20.4 |  | 26.6 (0.8) | 123 | 20.7 (2.4) | 16.2 (2.6) | 29.6 (3.8) | 18.4 | 13.8 – 23.1 |
| 17-18 years | 2 | 26.5 (0.06) | 19.3 | 18.7 – 19.8 | 24.9 (1.1) | 116 | 21.5 (2.7) | 17.5 (3.4) | 29.9 (5.3) | 19.9 | 14 – 25.7 |
| Values are depicted as mean (standard deviation) or median along with 95% confidence interval  UK = United Kingdom, ASMM = appendicular skeletal muscle mass, DXA = dual-energy x-ray absorptiometry, BMI = Body mass index, CI = confidence interval  ^1^McCarthy HD, Samani-Radia D, Jebb SA, Prentice AM. Skeletal muscle mass reference curves for children and adolescents. Pediatr Obes. 2014;9(4):249-259.  ^2^Webber CE, Barr RD. Age- and gender-dependent values of skeletal muscle mass in healthy children and adolescents. J Cachexia Sarcopenia Muscle. 2012;3(1):25-29. | | | | | | | | | | | |
